# The burden of acute kidney disease: an epidemiological review and importance of follow-up care

**DOI:** 10.1093/ckj/sfaf169

**Published:** 2025-05-27

**Authors:** Joana Gameiro, Beatriz Gouveia, José Agapito Fonseca, José António Lopes

**Affiliations:** Serviço de Nefrologia e Transplantação Renal, ULS Santa Maria, Lisboa, Portugal; Clínica Universitária de Nefrologia, Faculdade de Medicina da Universidade de Lisboa, Lisboa, Portugal; Clínica Universitária de Nefrologia, Faculdade de Medicina da Universidade de Lisboa, Lisboa, Portugal; Serviço de Nefrologia e Transplantação Renal, ULS Santa Maria, Lisboa, Portugal; Clínica Universitária de Nefrologia, Faculdade de Medicina da Universidade de Lisboa, Lisboa, Portugal; Serviço de Nefrologia e Transplantação Renal, ULS Santa Maria, Lisboa, Portugal; Clínica Universitária de Nefrologia, Faculdade de Medicina da Universidade de Lisboa, Lisboa, Portugal

**Keywords:** acute kidney disease, acute kidney injury, epidemiology, follow-up care, outcomes

## Abstract

Acute kidney injury (AKI) is a common and serious medical condition with increasing incidence over the past few decades. Patients with AKI undergo various clinical trajectories and they are at high risk of progression to chronic kidney disease (CKD). Acute kidney disease (AKD) defines the critical phase that is the transition from AKI to CKD. Given the increasing prevalence of AKD and its impact on patient prognosis in the long term, it is crucial that these patients receive multidisciplinary and effective follow-up care. This review aims to address the epidemiology, risk factors, and outcomes of AKD following AKI, providing deeper insights into this emerging health issue, with an emphasis on the importance of structured follow-up strategies for these patients.

## INTRODUCTION

Acute kidney injury (AKI) is a common and serious clinical illness defined by a sudden loss of kidney function that is defined by an increase in serum creatinine (SCr) levels or a decrease in urine output [1–4]. AKI is thought to affect >13 million individuals annually worldwide, with an incidence that varies from 3.2% to 20% in hospitalized patients and reaches up to 67% in critically ill patients [3]. Although mortality rates have declined, these patients undergo various clinical trajectories and these patients are at high risk for long-term adverse events, such as recurrent AKI episodes and hospital readmissions, progression to chronic kidney disease (CKD) or end-stage kidney disease, increased cardiovascular events, and long-term mortality [5–15].

These findings suggest that AKI and CKD are not always distinct entities and are likely to represent a continuum. Thus, it became crucial to define an entity for this phase, which is now known as acute kidney disease (AKD), which reflects the continuing pathological processes and adverse events developing after AKI [14]. This is a complex process involving cellular, vascular, and immune mechanisms that prevent kidney recovery and promote irreversible structural damage. Following AKI a maladaptive repair ensues, characterized by tubular epithelial cell cycle arrest and adoption of a profibrotic phenotype with extracellular matrix deposition, compensatory hypertrophy, and hyperfiltration leading to glomerular and tubular stress, mitochondrial dysfunction contributing to cellular injury, interstitial immune cell infiltration promoting persistent inflammation, and peritubular capillary loss resulting in hypoxia that furthers inflammation and fibrosis. With time, these mechanisms lead to tubulointerstitial fibrosis and glomerulosclerosis, in a cycle of injury and incomplete repair, thus culminating in progressive CKD [16, 17].

The concept of AKD was first suggested in the 2012 KDIGO AKI guidelines, but it was only in 2017 that the Acute Dialysis Quality Initiative (ADQI) 16 report proposed an alternative definition and classification of AKD [18, 19]. According to recent evidence, the incidence of AKD after AKI has increased, with a consistent pattern of doubling with every 10-year increase in age across all regions and years [20].

With this narrative review, we aim to address the epidemiology, risk factors, and outcomes of AKD after AKI, to provide a deeper insight of this arising health issue, with a focus on the importance of structured follow-up strategies for these patients.

## METHODS

We conducted a literature search of adult patients with AKD since 2017, using MEDLINE through the PubMed search engine with the MeSH terms: (i) AKD, diagnosis, (ii) AKD, epidemiology, (iii) AKD, risk factors, and (iv) AKD, long term, outcomes. The analyzed outcomes were development of CKD, cardiovascular events, and mortality.

### Acute kidney disease definition

The KDIGO guidelines define AKI as an abrupt decrease in kidney function that occurs over a period of 7 days or fewer, and CKD as abnormalities in kidney structure or function that persist for >90 days [19]. Still, there are patients that do not fulfill the criteria of either AKI or CKD [21]. These findings suggest that AKI and CKD are not always distinct entities and likely represent a continuum, as patients who have experienced an episode of AKI having an increased risk of developing either *de novo* CKD or worsening of pre-existing CKD [19]. Thus, it became crucial to define an entity for this period, which is now known as AKD [14].

**Figure 1:**
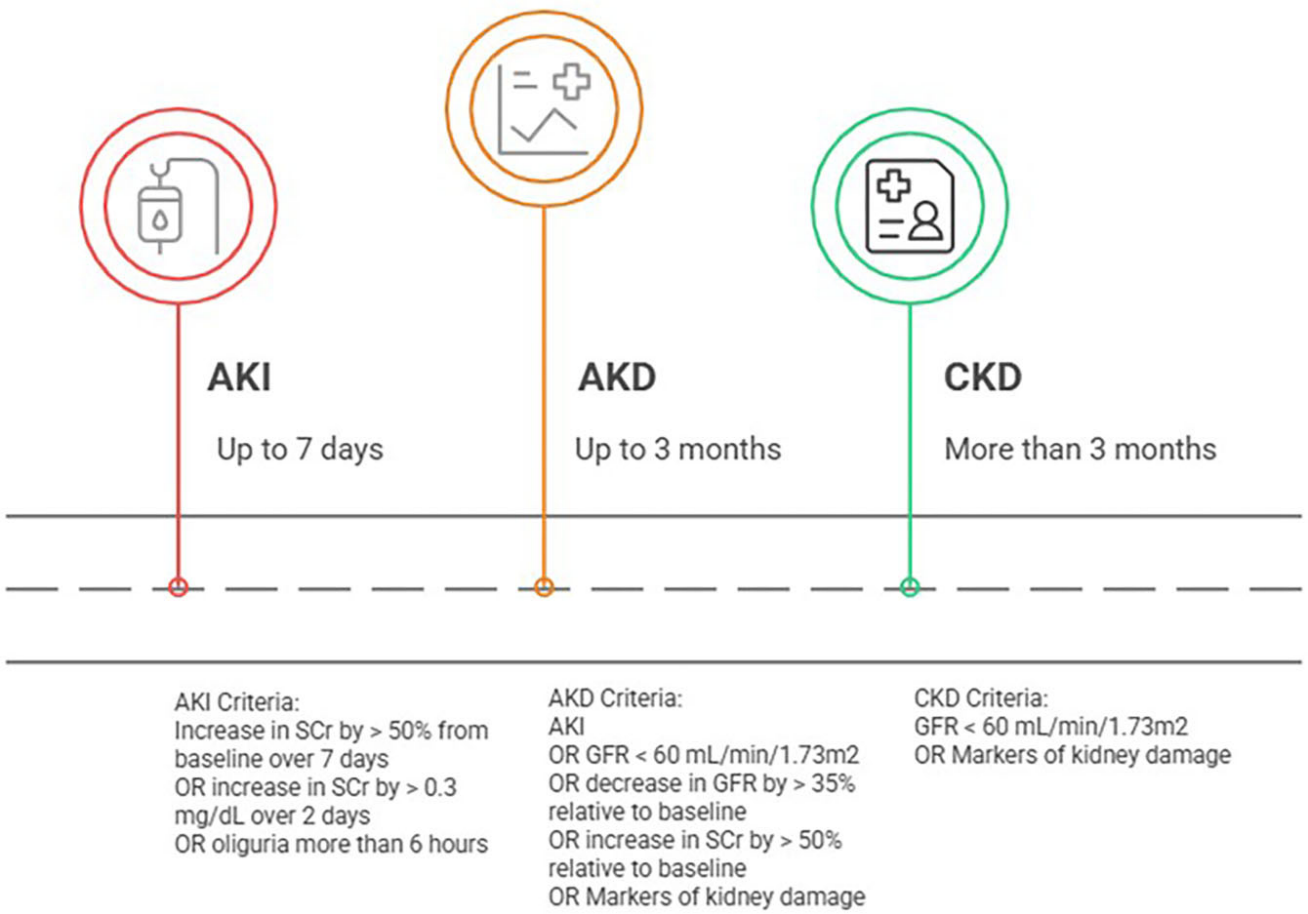
Definition and time frame for kidney diseases and disorders according to KDIGO guidelines.

**Figure 2:**
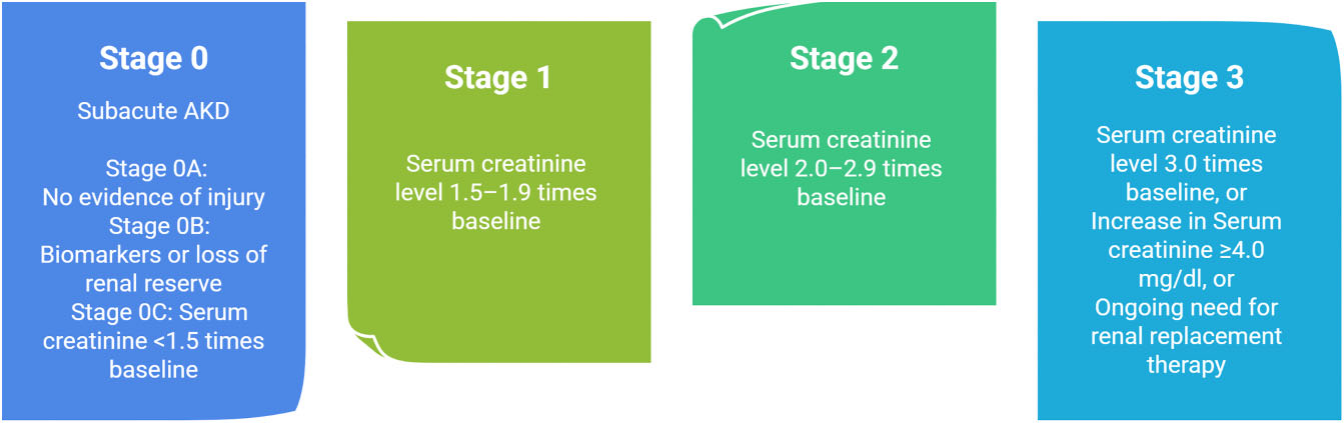
Severity of AKD as proposed by the ADQI 16th Workgroup.

Criteria for AKD were first proposed by the 2012 KDIGO AKI Workgroup and included any acute condition that affects kidney function such as AKI, a glomerular filtration rate (GFR) <60 ml/min/1.73 m^2^ for $< $3 months, a decrease in GFR by $\ge $35% or an increase in SCr of >50% for $< $3 months, or any kidney damage lasting <3 months (Fig. 1) [18, 21, 22]. This definition of AKD was designed to harmonize the criteria for the spectrum of clinical presentations of AKI, AKD, and CKD. In 2017, the 16th ADQI Workgroup proposed a new definition for AKD, as a condition in which AKI stage 1 or greater, as defined by KDIGO, is present ≥7 days after an AKI initiating event [19]. Although in most cases, an AKI initiating event can be identified, it is not required to diagnose AKD [18, 19, 22, 23]. The Workgroup also proposed an AKI-based staging system for AKD post-AKI episode (Fig. 2) [19].

With this updated AKD classification, clinicians can now identify an important population of patients with evolving kidney disease who might not fulfill strict criteria for AKI or CKD. Furthermore, this new definition highlights that the process of AKD can include AKI and that it includes the process of recovery or worsening until the criteria for CKD is reached [19].

In the absence of specifics on the duration of AKI, such as patients with community-acquired AKI in whom baseline kidney function is unknown, or in patients with AKD without AKI, an approach based on GFR, once stability is achieved, is more appropriate. The Workgroup also suggested adding albuminuria categories to GFR for AKD severity staging [18, 22]. Even though staging and classifying AKD is highly desirable, further evidence is required before the approach is standardized [18, 19, 22].

It is possible that some biomarkers used in either AKI or CKD might be useful in further categorizing AKD [24–27]. In Chen *et al.*’s study of 269 patients admitted to a Coronary Care Unit, a multivariate analysis identified that higher levels of serum interleukin 18 (IL-18) were predictive of AKD (*P* = .015). According to this study, higher levels of serum IL-18 might indicate more severe renal parenchymal injury, prolonging the recovery time from AKI and leading to AKD [28]. Another study of 209 patients with infective endocarditis identified that patients with AKD had significantly higher levels of cystatin C (1.7 vs 1.3 mg/l; *P* = .035) and neutrophil gelatinase-associated lipocalin (NGAL) (19 vs 1.9 ng/ml; *P* = .05) when compared with patients without AKD [29]. Similarly, NGAL was also predictive of AKD in a recent randomized controlled trial of 1341 patients with septic shock (AUROC 0.74) [30]. Based on these results, urinary NGAL and serum cystatin C might be considered as AKD markers, however, there are insufficient data to recommend the routine use of novel biomarkers for AKD staging.

Aside from biomarkers, other additional tools, such as imaging techniques could help diagnose AKD earlier. An observational study demonstrated that renal scintigraphy could identify patients with higher risk of persistent renal failure after AKI with a high specificity (90%) but a low sensitivity (35%), although its potential clinical applications need to be tested in prospective studies [31]. Doppler ultrasound might also be helpful in identifying AKD patients, as an elevated Doppler-based renal resistive index has been reported as an accurate predictor of persistent AKI in critically ill patients [32]. Additionally, magnetic resonance imaging has also been demonstrated to effectively identify persistent abnormalities in patients 3 months after AKI, such as reduced cortical perfusion, despite recovered kidney function when assessed by serum creatine levels [33, 34]. More research is underway to better identify and classify AKD patients.

### Epidemiology

In recent years, many groups have attempted to characterize the epidemiology of AKD after AKI, with reports ranging from 1% to 33%.

In a large comprehensive population-based cohort study by Wang *et al.*, which included 56 906 patients with community and hospital-acquired AKI, 18 773 (33%) patients progressed to AKD [35]. Another study of 62 977 hospitalized adults with preserved baseline kidney function, reported that 906 (1%) patients had AKD after AKI [36]. The considerably lower incidence rate of AKD in this study can be explained by the younger age of this cohort and the bias in selection of patients.

According to a meta-analysis of 21 studies, which included >1 million patients, 26.11% of hospitalized patients progressed to AKD after an AKI episode [37]. The incidence of AKD also varied according to the clinical scenario, namely it was nearly 40% in patients with AKI and myocardial infarction, 35.9% in patients with AKI after surgery, and 15.6% in patients after severe malaria-related AKI [37]. This was influenced not only by the AKI setting, but also by the severity of AKI but also by patient characteristics, such as age and comorbidities. Additionally, methodological differences in the definitions of AKI and AKD may further contribute to this variability.

Indeed, the incidence of AKD according to surgical setting can vary greatly. The incidence of AKD after AKI in the context of vascular and cardiac surgery have been reported to be similar, ranging mainly from 12% to 30%, and lower at around 8% in orthopedic procedures [38–41]. In Chen *et al.*’s study of 328 patients who developed AKI after surgery for acute type A aortic dissection, 29.9% progressed to AKD [38]. Similarly, a study of 5127 patients undergoing cardiac surgery reported that, among the patients with AKI, 273 (27.5%) developed AKD [39]. However, in another study of 3605 patients undergoing cardiac surgery, 74.1% of patients with persistent AKI progressed to AKD [40]. According to Meersch *et al.*’s study, out of 9510 postoperative patients without pre-existing CKD, 9.9% developed AKD after 7 days, and among these, only 34.1% of patients had a documented episode of postoperative AKI [41]. The incidence of AKD significantly increased with the severity and duration of AKI [41]. This highlights the impact of not only the presence of AKI and its severity but also duration of AKI in the progression to AKD.

Regarding critically ill patients, the incidence of AKD seems to be higher, possibly explained by the higher severity of AKI and of the clinical condition. In a study of 1231 AKI patients with sepsis 46.9% of patients developed AKD [42]. In another single-center retrospective analysis of 256 patients with septic AKI, 53.9% developed AKD [43].

### Risk factors

Considering the rising incidence of AKD, it is crucial to recognize the risk factors associated with AKD development after AKI (Table 1).

**Table 1:** Summary of the risk factors associated with AKD development.

Author/year	Number of patients	Study design	AKD criteria/incidence	Risk factors for AKD
Meersch *et al.* 2024	9 510	Prospective	eGFR < 60 ml/min/1.73 m^2^ 9.9%	Female sex (OR male sex: 0.45; 95% CI 0.38–0.52; *P* < .001) Advanced age (OR 1.05; 95% CI 1.04–1.06; *P* < .001) Early postoperative AKI (OR 2.64; 95% CI 2.21–3.15; *P* < .001) Comorbidities • Hypertension (OR 1.32; 95% CI 1.11–1.58; *P =* .002) • Atrial fibrillation (OR 1.44; 95% CI 1.14–1.79; *P =* .002) • Myocardial infarction (OR 1.25; 95% CI 1.02–1.54; *P =* .034) • Peripheral vascular disease (OR 1.30; 95% CI 1.02–1.65; *P =* .030) Emergency procedures (OR 1.73; 95% CI 1.19–2.47; *P =* .003) Intraoperative nephrotoxic agents • Vancomycin (OR 2.06; 95% CI 1.07–3.87; *P =* .027) • Cyclosporine or tacrolimus (OR 6.32; 95% CI 1.65–21.72; *P =* .004) Postoperative nephrotoxic agents • Aminoglycosides (OR 2.54, 95% CI 1.31–4.65; *P =* .004) Postoperative complications • Pneumonia (OR 2.07; 95% CI 2.21–3.15; *P* = .006)
Chen *et al.* 2023	328	Clinical trial	ADQI 2017 29.9%	Comorbidities • Hypertension (OR: 2.302; 95% CI: 1.194–4.438; *P* = .013) Lung infection after surgery (OR: 3.278; 95% CI: 1.794–5.989; *P* < .001) Baseline SCr (OR: 1.109; 95% CI: 1.009–1.028; *P* < .001) Cys-C (OR: 1.469; 95% CI: 1.145–1.883; *P* = .002) AKI severity: AKI stage 2 (OR: 3.032; 95% CI: 1.377–6.677; *P* = .006) AKI stage 3 (OR: 4.001; 95% CI: 1.755–9.122; *P* = .001)
Pan *et al.* 2023	377 823	Retrospective	ADQI 2017 34.4%	Female sex (OR male sex: 0.78, 95% CI 0.72–0.85; *P* < .001) Advanced age ($\ge $70 years) (OR 1.16, 95% CI 1.06–1.27; *P* < .001) Comorbidities • Underlying early CKD stage 1–2 (OR 1.46, 95% CI 1.23–1.73; *P* < .001) • Chronic liver disease (OR 1.43, 95% CI 1.27–1.61; *P* < .001) Malignancy (OR 1.30, 95% CI 1.14–1.47; *P* < .001) AKI severity: AKI stage 2 (OR 2.15, 95% CI 1.92–2.41, *P* < .001), AKI stage 3 (OR 1.91, 95% CI 1.72–2.12, *P* < .001) Use of emergency hemodialysis (OR 1.29, 95% CI 1.04–1.59; *P =* .019) Use of dopamine (OR 1.14, 95% CI 1.03–1.25; *P =* .009)
Xu *et al.* 2023	71 041	Retrospective	ADQI 2017 14.4%	Comorbidities • Malignancy (OR: 1.26; 95% CI: 1.16–1.36; *P <* .01) Hypoproteinemia (OR: 1.09; 95% CI: 1.02–1.17; *P <* .05)
Zhang *et al.* 2023	1 062	Retrospective	ADQI 2017 3.9%	Advanced age (HR: 1.078; 95% CI: 1.029–1.112; *P* < .001) Baseline eGFR (HR: 1.015; 95% CI: 1.001–1.030; *P* < .001) RENAL score (HR: 1.612; 95% CI: 1.067–2.437; *P* = .023) Ischemia time >30 min (HR: 7.284; 95% CI: 2.210–23.999; *P* = .001) Intraoperative blood loss >300 ml (HR: 8.641; 95% CI: 2.751–27.171; *P* < .001)
Chen *et al.* 2022	7 519	Retrospective	ADQI 2017 21.2%	Comorbidities • Diabetes mellitus (OR: 1.15; 95% CI: 1.01–1.31; *P* = .030) • CKD (OR: 1.54; 95% CI: 1.34–1.76; *P* < .001) AKI severity: AKI stage 1 (OR: 2.15; 95% CI: 1.71–2.70; *P* < .001), AKI stage 2/3 (OR: 2.13; 95% CI: 1.62–2.82; *P* < .001) Laboratory values • Hemoglobin <10 g/dl (OR: 1.46; 95% CI: 1.27–1.68; *P* < .001) • Albumin <3.5 g/dl (OR: 1.48; 95% CI: 1.30–1.68; *P* < .001) • BNP per 100 pg/ml (OR: 1.01; 95% CI: 1.002–1.01; *P* = .010) Medication • Inotropes (OR: 1.28; 95% CI: 1.11–1.48; *P* = .001) • IV furosemide use/cumulative dosage per 10 mg (OR: 1.04; 95% CI: 1.03–1.06; *P* < .001)
Hsu *et al.* 2022	310	Retrospective	ADQI 2017 77.1%	Use of offending drugs (*P* = .038) AKI severity (*P* < .001) RRT requirement within the 1st week (*P* = .057)
Patidar *et al.* 2022	6 250	Retrospective	ADQI 2017 31.6%	Comorbidities • CKD (OR: 3.14; 95% CI: 2.49–3.96; *P* < .001) • Ascites (OR: 1.60; 95% CI: 1.27–2.00; *P* < .001) • Obesity (OR: 1.48; 95% CI: 1.20–1.86; *P* = .001) AKI severity: AKI stages 2/3 (OR: 9.37; 95% CI: 7.02–12.50; *p* < .001) AKI setting: Community-acquired AKI (OR: 1.63; 95% CI: 1.25–2.14; *P* < .001) Lower serum albumin at time of AKI (OR: 1.37; 95% CI: 1.07–1.75; *P* = .013) Lower MAP at time of AKI (per 1 mmHg decrease, OR: 1.01; 95% CI: 1.00–1.03; *P* < .001)
Sarwal *et al.* 2022	1 074	Retrospective	ADQI 2017 28.2%	Comorbidities • Congestive heart failure (OR: 4.59; 95% CI: 1.76–11.78; *P* = .002) Baseline CKD (stage ≥ 3) (OR: 3.23; 95% CI: 1.59–6.56; *P* = .001) AKI on admission (stage 3) (OR: 2.71; 95% CI: 1.14–6.46; *P* = .025) Ongoing diarrhea during the hospital course (OR: 3.19; 95% CI: 1.02–9.96; *P* = .025)
Wang *et al.* 2022	56 906	Retrospective	ADQI 2017 33%	Female sex (OR male sex: 0.86; 95% CI: 0.76–0.97; *P* = .016) Comorbidities • Malignancy (OR: 1.22; 95% CI: 1.17–1.28; *P* < .001) • Chronic heart failure (OR: 1.33; 95% CI: 1.24–1.42; *P* < .001) AKI severity: AKI stage 2 (OR: 1.23; 95% CI: 1.15–1.30; *P* < .001), AKI stage 3 (OR: 2.40; 95% CI: 2.21–2.61; *P* < .001) AKI setting: Community-managed AKI (OR: 2.34; 95% CI: 2.17–2.53; *P* < .001), Hospital-acquired AKI (OR: 1.91; 95% CI: 1.82–2.00; *P* < .001) • Recent use of loop diuretics (OR: 1.12; 95% CI: 1.06–1.18; *P* < .001)
Cho *et al.* 2021	1 386	Retrospective	KDIGO 2012 46.3%	Advanced age (OR: 1.05; 95% CI: 1.03–1.08; *P* < .001) Low pre-operative albumin level (OR: 0.45; 95% CI: 0.29–0.68; *P* < .001) Postoperative AKI (OR: 2.33; 95% CI: 1.45–3.73; *P* < .001) AKI severity: AKI stage 1 (OR: 3.02; 95% CI: 1.82–5.02; *P* < .001), AKI stage 2 (OR: 5.20; 95% CI: 2.26–12.00; *P* < .001), AKI stage 3 (OR: 6.20; 95% CI: 2.65–14.51; *P* < 0.001) Pre-existing renal dysfunction (OR: 3.51; 95% CI: 2.33–5.28; *P* < .001)
Marques *et al.* 2021	544	Retrospective	ADQI 2017 25.7%	Comorbidities • Hypertension (OR: 2.95; 95% CI: 1.36–6.38; *P* = .006) • CKD (OR: 6.17; 95% CI: 2.56–19.43; *P* < .001) Nephrotoxin exposure (OR: 6.76; 95% CI: 2.82–16.17; *P* < .001)
Nagata *et al.* 2021	76 090	Retrospective	ADQI 2017 1.37%	Persistent AKI (HR: 6.26; 95% CI: 4.66–8.40; *P* < .001)
See *et al.* 2021	62 977	Retrospective	KDIGO 2012 2%	Advanced age (OR: 1.36; 95% CI: 1.31–1.41; *P* < .001) Smoking (OR: 1.53; 95% CI: 1.35–1.72; *P* < .001) Comorbidities • Diabetes (OR: 1.58; 95% CI: 1.39–1.80; *P* < .001) • Hypertension (OR: 3.05; 95% CI: 2.66–3.50; *P* < .001) • Cerebrovascular disease (OR: 1.86; 95% CI: 1.54–2.23; *P* < .001) • Peripheral vascular disease (OR: 2.99; 95% CI: 2.48–3.64; *P* < .001) • Chronic heart failure (OR: 4.83; 95% CI: 4.09–5.69; *P* < .001) • Liver disease (OR: 1.34; 95% CI: 1.13–1.60; *P* = .001) • Hematological disease (OR: 2.58; 95% CI: 1.85–3.62; *P* < .001) • Malignancy: Non-metastatic (OR: 2.70; 95% CI: 2.23–3.27; *P* < .001), Metastatic (OR: 3.10; 95% CI: 2.49–3.87; *P* < .001)
Tonon *et al.* 2021	272	Prospective	KDIGO 2012 29.4%	Advanced age (HR: 1.05; 95% CI: 1.02–1.07, *P* < .001) Higher Child Turcotte Pugh score (HR: 1.24; 95% CI: 1.12–1.39, *P* < .001) -Higher Model for End-Stage Liver Disease score (HR: 1.10; 95% CI: 1.04–1.16, *P* = .001)
Yan *et al.* 2021	34 709	Retrospective	ADQI 2017 45.4%	AKI severity: AKI stage 2 (OR: 6.87; 95% CI: 4.71–10.01; *P* < .001), AKI stage 3 (OR: 34.1; 95% CI: 23.30–49.91; *P* < .001) AKI setting: hospital-acquired AKI vs community-acquired AKI (OR: 1.80; 95% CI: 1.42–2.27; *P* < .001) AKI classification • Pre-renal (OR: 1.28; 95% CI: 0.81–2.03; *P* = .282) • Intrinsic-renal (OR: 1.63; 95% CI: 1.011–2.62; *P* = .046) • Post-renal (OR: 0.75; 95% CI: 0.42–1.34; *P* = .336) Proteinuria (OR: 1.45; 95% CI: 1.10–1.90; *P* = .008) Anemia (OR: 1.43; 95% CI: 1.15–1.78; *P* = .002) Hyperuricemia (OR: 1.33; 95% CI: 1.07–1.65; *P* = .012) Organ failure (OR: 1.13; 95% CI: 1.04–1.23; *P* = .003) Higher Charlson Comorbidity Index (OR: 1.10; 95% CI: 1.05–1.15; *P* < .001)
Chen *et al.* 2020	269	Retrospective	ADQI 2017 47.6%	Advanced age (OR: 1.034; 95% CI: 1.007–1.063; *P* = .014) Higher serum IL-18 (OR: 1.002; 95% CI: 1.000–1.004; *P* = .015)
Xiao *et al.* 2020	34 709	Retrospective	ADQI 2017 3.92%	Advanced age ($\ge $65 years) (OR: 1.758; 95% CI: 1.286–2.404; *P* < .001) Female sex (OR: 0.545; 95% CI: 0.405–0.732; *P* < .001) Hepatorenal syndrome (OR: 8.205; 95% CI: 2.710–24.842; *P* < .001) Oliguria or anuria (OR: 3.104; 95% CI: 1.997–4.826; *P* < .001) Respiratory failure (OR: 1.976; 95% CI: 1.281–3.049; *P* = .002) BUN $\ge $ 7.14) (OR: 1.933; 95% CI: 1.414–2.642; *P* < .001) AKI severity: AKI stage 2 (OR: 3.289; 95% CI: 2.399–4.508; *P* < .001), AKI stage 3 (OR: 18.787; 95% CI: 12.813–27.546; *P* < .001)
Kofman *et al.* 2019	225	Retrospective	ADQI 2017 36%	Advanced age (OR: 1.02, 95% CI: 0.98–1.04, *P* = .09) AKI severity (OR: 1.53; 95% CI: 1.13–2.06, *P* = .005)
Mizuguchi *et al.* 2018	10 234	Retrospective	KDIGO 2012 9.2%	AKI severity: AKI stage 1 (RR: 2.31; 95% CI: 1.23–4.33; *P* = .009), AKI stage 2 (RR: 9.36; 95% CI: 4.74–18.5; *P* < .001), AKI stage 3 (RR: 22.9; 95% CI: 13.9–37.6; *P* < .001)

BNP, B-type natriuretic peptide; BUN, blood urea nitrogen; CI, confidence interval; Cys-C, cystatin c; eGFR, estimated GFR; HZ, hazard ratio; IV, intravenous; MAP, mean arterial pressure; OR, odds ratio; RR, relative risk

Recent research indicates that older age increases the risk of developing AKD, with odds ratios ranging from 1.05 (95% CI 1.04–1.06, *P* < .001) to 1.758 (95% CI 1.286–2.404, *P* < .001) [37, 41, 44–49]. With aging, the kidneys undergo morphological, anatomical, and functional changes that lead to lower glomerular filtration rates. Additionally, older individuals often experience a higher burden of comorbidities and are more exposed to nephrotoxic medications, all of which increase the susceptibility of developing AKD after AKI [49, 50].

The presence of CKD is one of the most important risk factors, which increases the risk of AKD after AKI up to six times [44, 45, 51–55]. Pan *et al.*, reported that in 11 045 AKI survivors, CKD was associated with a 1.46-fold higher risk of AKD (95% CI 1.23–1.73; *P* < .001) [44]. In CKD patients, the tubulointerstitial lesions render the kidneys more vulnerable to various acute insults, which lead to lower rates of renal function recovery after AKI, therefore increasing the risk of AKD [56]. Moreover, several studies suggest that other comorbidities, such as hypertension (OR: 3.05; 95% CI 2.66–3.50; *P* < .001), chronic heart failure (OR: 4.83; 95% CI 4.09–5.69; *P* < .001), diabetes mellitus (OR: 1.58; 95% CI 1.39–1.80; *P* < .001), liver disease (OR: 1.43; 95% CI 1.27–1.61; *P* < .001), and malignancy (OR: 3.10; 95% CI 2.49–3.87; *P* < .001), also contribute to the development of AKD [38, 39, 42, 45, 51, 54, 57–59].

The severity of AKI also has a significant role in the progression to AKD [39, 42, 45, 47, 50–53, 55, 58–60]. In fact, Yan *et al.*’s study demonstrated that AKI stage 2 and stage 3 were major risk factors for AKD compared to AKI stage 1, with stage 3 increasing the risk of AKD up to 34 times (OR: 34.1; 95% CI 23.30–49.91; *P* < .001) [59]. This was also reported in a study by Hsu *et al.*, in which more severe AKI was associated with AKD severity (OR: 6.214, 95% CI 2.658–14.526, *P* < .001) [58].

The duration of AKI is also a critical factor in AKD development. Recent studies have shown that persistent AKI, which lasts >48 h from its onset, has been associated with a higher progression to AKD than transient AKI. [19, 60, 61] According to Nagata *et al.*, renal function decline is more pronounced following persistent AKI (HR: 6.26; 95% CI 4.66–8.40, *P* < .001). This supports the hypothesis that persistent AKI might be associated with more severe structural kidney damage, which could explain why there is a higher risk of progression to AKD [61].

Finally, the administration of nephrotoxic medications contributes to the development of AKD. In a retrospective study of 310 hospitalized AKD patients, the use of nephrotoxic drugs was associated with more than three times the risk of AKD stage 3, and more than twice the risk of AKD stage 2 (AKD stage 2 OR: 2.314, 95% CI 1.049–5.107, *P* = .038; AKD stage 3 OR 3.132, 95% CI 1.304–7.526, *P* = .011) [58]. By worsening kidney disfunction, these drugs delay kidney recovery and ultimately contribute to AKD.

### Outcomes

AKD has consistently been associated with worse patient outcomes. Persistent kidney dysfunction following AKI is associated with an increased risk of progression to CKD, End-Stage Kidney Disease (ESKD), cardiovascular complications, and mortality [22, 36] (Table 2).

**Table 2:** Summary of AKD outcomes.

Author/year	Number of patients	Study design/AKD criteria	Follow-up	Renal outcomes	MACE	Mortality
Jensen *et al.* 2024	169 582	Retrospective ADQI 2017	2.3 years	CKD • HR 1.36, 95% CI 1.30–1.41 ESKD • HR 1.56, 95% CI 1.44–1.69	Overall cardiovascular disease • HR 1.01, 95% CI 0.96–1.07 Heart failure • HR 1.09, 95% CI 1.03–1.15 Ischemic heart disease • HR 1.11, 95% CI 1.03–1.19 Peripheral artery disease • HR 1.10, 95% CI 1.02–1.17	
Pan *et al.* 2024	6 703	Retrospective ADQI 2017	1.2 years	ESKD 16.7%	11.1%	All-cause mortality 28.3% • Post-AKD CKD stage 3: HR 1.19, 95% CI 1.02–1.38 • Post-AKD CKD stage 4: HR 1.58, 95% CI, 1.32–1.89 • Post-AKD CKD stage 5: HR 1.56, 95% CI, 1.26–1.93
Su *et al.* 2023	1 114 012	Meta-analysis		CKD • AKD vs NKD: 37.2% vs 7.45% OR 4.22 95% CI 2.79–6.39, *P* < .001 ESKD • AKD vs NKD: 1.3% vs 0.14% OR 6.58 95% CI 3.75–11.55, *P* < .001		AKD vs NKD: 26.54% vs 7.78%; OR 3.62, 95% CI 2.64–4.95, *P* < .001
Xu *et al.* 2023	71 041	Retrospective ADQI 2017	14 months	CKD • 10.5%; HR 2.49, 95% CI 2.37–2.62, *P <* .05		16%; HR 4.51, 95% CI 4.32–4.71, *P <* .05
Chen *et al.* 2022	7 519	Retrospective ADQI 2017	5 years	MAKE • HR 1.30, 95% CI 1.21–1.40	Heart failure hospitalization • HR 1.20, 95% CI 1.07–1.34	HR 1.32, 95% CI 1.17–1.49
Esposito *et al.* 2022	63	Retrospective KDIGO 2020	3 months	CKD • AKD stage 2–3: OR 14, 95% IC 1.4–134, *P* = .013		
Liu *et al.* 2022	9 223	Observational KDIGO 2012	5.4 years			AKD vs Non-AKD: 24.8% vs 15.4%; HR 1.57, 95% CI: 1.39–1.78, *P* < .001
Patidar *et al.* 2022	6 250	Retrospective ADQI 2017	180 days	CKD • AKD vs Non-AKD: 64.0% vs 30.7% HR 2.52; 95% CI 2.01–3.15, *P* < .001		AKD vs Non-AKD • At 90-days: 31.7% vs 20.1%, *P* < .001 HR 1.37, 95% CI 1.14–1.65, *P* = .001 • At 180-days: 36.0% vs. 24.0%, *P* < .001 HR 1.37, 95% CI 1.14–1.64, *P* = .001
Wang *et al.* 2022	56 906	Retrospective ADQI 2017	2.1 years	CKD • HR 2.21, 95% CI 1.91–2.57		HR 1.20, 95% CI 1.13–1.26
Zhou *et al.* 2022	5 065	Prospective KDIGO 2012	1 year			HR 2.67, 95% CI 1.27–5.61, *P* = .01
Flannery *et al.* 2021	6 290	Retrospective ADQI 2017	14.2 months	MAKE • Stage 0C AKD vs Stage 0A AKD: HR 1.74, 95% CI 1.32–2.29 • Stage $\ge $1 AKD vs Stage 0A AKD: HR 3.25, 95% CI 2.52–4.20 • Stage $\ge $1 AKD vs Stage 0C AKD: HR 1.87, 95% CI 1.44–2.43		
Yan *et al.* 2021	2 556	Retrospective ADQI 2017	1 year	MAKE • AKD stage 1: OR 2.36, 95% CI 1.66–3.36, *P* < .001 • AKD stage 2–3: OR 31.35, 95% CI 23.42–41.98, *P* < .001	-	30-days mortality • AKD stage 2–3: OR 2.52, 95% CI 1.86–3.42, *P* < .001 1-year mortality • AKD stage 2–3: OR 2.08, 95% CI 1.67–2.59, *P* < .001
Matsuura *et al.* 2020	3 605	Retrospective ADQI 2017	2 years	eGFR decline • 30% eGFR decline: OR 1.79, 95% CI 1.30–2.40 • 40% eGFR decline: OR 2.62, 95% CI 1.81–3.75 • 50% eGFR: OR 3.56, 95% CI 2.24–5.57		90-day mortality • AKD vs Non-AKD: OR 63.0, 95% CI 27.9–180.6
Lin *et al.* 2018	6 306	Retrospective ADQI 2017	5.99 years			HR 1.27, 95% CI 1.18–1.36, *P* < .001

AKD often results from an incomplete recovery from AKI, characterized by ongoing structural or functional kidney abnormalities, which can trigger a maladaptive repair process that leads to tubulointerstitial fibrosis and progressive nephron loss, ultimately resulting in CKD [36, 37]. Following a recent meta-analysis by Su *et al.*, AKD patients had a higher incidence rate of CKD (OR: 4.22, 95% CI 2.79–6.39, *P* < .01). [37] According to a retrospective study of 56 906 patients with community or hospital-acquired AKI, progression to AKD was associated with a 2-fold increased risk of developing *de novo* CKD over a median 2-year follow-up period (HR: 2.21, 95% CI 1.91–2.57) [59]. Similarly, a study of 71 041 hospitalized patients, who were followed for a median of 14 months, reported that AKD increased the risk of CKD up to 2.49 times (HR: 2.49, 95% CI 2.37–2.62, *P* < .05) [57]. A significantly higher risk of progression to CKD was reported in a retrospective study by Esposito *et al.* of 63 patients with AKI requiring hemodialysis, in which the risk was up to 14 times higher (OR: 14, 95% CI 1.4–134, *P* = .013), which can be attributed to the increased severity of this cohort [62]. Indeed, a higher severity of AKD also appears to correlate with the risk of CKD development, as patients with stage 1 or greater AKD carry a higher risk (HR: 1.87; 95% CI: 1.44–2.43) [42]. Recovery timing is also an independent predictor of progression to CKD. This was recently demonstrated in Wang *et al.*’s study, in which the risk of developing CKD correlated with longer recovery timing over the first month following AKI [35]. In fact, longer recovery timing is associated with persistent inflammation, prolonged renin-angiotensin system activation that results in long-term hypertension and fibrosis, all of which contribute to the AKI to AKD to CKD transition [56].

Patients with AKD also have a higher risk of progressing to ESKD. In a recent systematic review, AKD was associated with a risk of progression to ESKD of 1.3% over 7.7 years of follow-up (OR: 6.58, 95% CI 3.75–11.55, *P* < .001), although study heterogeneity was high [37]. In fact, in a retrospective cohort study of 2556 hospitalized AKI patients, AKD was associated with a higher risk of persistent renal dysfunction and renal replacement therapy (RRT) over 30 days, namely AKD stages 2–3 were linked to an 18 times higher risk of RRT (OR: 18.86, 95% CI 5.74–62.01, *P* < .001) [59]. In line with these findings, a study by Flannery *et al.* of 6290 patients admitted to the ICU with sepsis, reported a significant association between AKD and the composite of CKD incidence or progression, RRT, and mortality [42]. Moreover, these outcomes correlated with AKD severity (Stage 0C AKD HR: 1.74, 95% CI 1.32–2.29; Stage $\ge $1 AKD HR: 3.25, 95% CI 2.52–4.20; Stage $\ge $1 AKD HR: 1.87, 95% CI 1.44–2.43), thus reinforcing the importance of assessing AKD severity when evaluating the risk of long-term kidney outcomes [42].

Recent studies have also demonstrated an increased incidence of cardiovascular events after AKD [50, 63, 64]. In a study of 7519 patients with acute heart failure, AKD patients had an increased rate of heart failure hospitalization over a 5 year follow-up period (HR: 1.20, 95% CI 1.07–1.34) [50]. Another study by Zhou *et al.* including 5065 patients with ischemic stroke reported that AKD development increased the risk of post-stroke disability over a 1-year follow-up (OR: 1.60; 95% CI: 1.04–2.44, *P* = .03), thereby suggesting that AKD might affect stroke recovery [63]. More recently, a retrospective cohort study, which included 169 582 AKI patients, concluded that AKD was associated with higher rate of heart failure (HR: 1.09, 95% CI 1.03–1.15), ischemic heart disease (HR: 1.11, 95% CI 1.03–1.19), and peripheral artery disease (HR: 1.10, 95% CI 1.02–1.17) over a 2-year follow-up [64].

Cardiovascular disease and CKD are closely interconnected, with CKD significantly increasing the risk of cardiovascular morbidity and mortality. The increased risk of cardiovascular disease after AKD could, therefore, be associated with the progression to CKD [40]. Cardiovascular risk factors such as hypertension and diabetes are highly prevalent in patients with CKD and contribute to kidney disease progression and to cardiovascular disease. The presence of uremic toxins and volume overload, further exacerbate cardiovascular risk. Additionally, overactivation of the renin–angiotensin–aldosterone pathway and sympathetic system, systemic inflammation, endothelial dysfunction, uremic state and disturbances in calcium-phosphorus metabolism in CKD patients trigger mechanisms that ultimately result in hypertension, myocardial remodeling, atherosclerosis and vascular calcifications [65, 66].

Patients with AKD also have a significantly higher risk of mortality, with a reported mortality rate of 26.5% (HR:1.39, 95% CI 1.25–1.55, *P* < .001) in a recent systematic review [37]. According to Liu *et al.*’s study of 9223 patients who underwent coronary angiography, AKD was associated with a 1.57-fold increased risk of all-cause mortality over a mean of 5.4 years (*P* < .001) [67]. This has been consistent throughout literature. In a study of 6250 hospitalized patients with cirrhosis and AKI, AKD patients had an increased risk of mortality at 90 and 180 days (HR: 1.37, 95% CI 1.14–1.64, *P* = .001) [51]. Similarly, another study of 6306 patients with AKI requiring dialysis the risk of mortality was 1.27 times higher in AKD patients over almost 6 years of follow-up (HR: 1.27, 95% CI 1.18–1.36, *P* < .001) [68]. A significantly higher risk of mortality was reported in 3605 patients admitted to intensive care units after cardiac surgery over a 2-year follow-up period (OR: 63.0, 95% CI 27.9–180.6), which also highlights the impact of illness severity in long-term outcomes [40].

This increased mortality risk might be attributed to the ongoing kidney dysfunction and systemic inflammation, leading to metabolic dysregulations, fluid overload, and decreased toxin clearance, all of which amplify the risk of cardiovascular complications and multiorgan failure. Furthermore, AKD occurs in patients with severe acute illnesses or comorbidities, which contribute to the mortality risk [69].

### Importance of nephrology follow-up

AKD marks a critical transitional period for patients who have experienced an episode of AKI, thus presenting the perfect opportunity to block the AKI-CKD transition and improve long-term outcomes [19, 22]. Therefore, it is crucial to enhance the follow-up care provided to these patients.

It is recommended that patient assessment after discharge should occur within 3 months based on the degree of GFR decline and the level of albuminuria, as nephrology evaluation within 90 days of hospital discharge after AKI has been associated with better survival in previous studies [22, 69–71]. Despite these recommendations, the proportion of patients who receive structured follow-up care after an AKI episode remains disappointingly small. In a retrospective cohort study of 20 260 patients with AKI, only 37.3% received nephrology follow-up during the AKD period, which was defined as the 7–90 days after index hospital discharge [72]. This was similar in another report from North America, in which only 25% survivors of AKI had an outpatient creatinine and urine protein measurement at 3 months [73]. Thus, there is an urgent need to promptly address the gap between the recommendations of follow-up care after AKI and actual clinical practice.

The use of automated alerts integrated into electronic health records can prompt clinicians to arrange follow-up and reinforce guideline-based care. Evidence suggests that these alerts improved the recognition of AKI and prompted changes in clinical practice, although it is yet to be proved that these have an impact on patient outcomes [74].

Several barriers to follow-up care have been identified including lack of recognition of AKI and AKD and its impact on outcomes by health care providers and patients, fragmented transitions of care between hospital and primary care, limited access to nephrology services, and lack of evidence for effective interventions to improve outcomes after AKI. The FUSION trial was only able to enroll 26% of eligible post-AKI patients as they were reluctant to have more doctor appointments [75]. Similarly, a study of 98 AKI stage 3 survivors also had difficulties in recruiting patients for follow-up after hospitalization, although the authors were still able to report that implementing comprehensive care in these patients resulted in a lower urine albumin–creatinine ratio (*P* = .036) and better blood pressure control (*P* = .006). Despite this, no difference in kidney outcomes was found at the 1-year follow-up [76].

More recently, Silver *et al.* demonstrated that a specialized AKI follow-up clinic was associated with lower mortality risk (HR: 0.71, 95% CI 0.55–0.91) and increased prescriptions of beta-blockers and statins, although renin–angiotensin–aldosterone system inhibitor (RAASi) prescription was not significantly increased. Also, the risk of adverse renal outcomes was not reduced, but the mean follow-up was only ∼2 years [77]. In a randomized controlled trial of 98 AKI patients, those who had comprehensive follow-up care had lower urine albumin–creatinine ratio (*P* = .036) and better blood pressure control (*P* = .006). Although there were no differences in renal outcomes in 1 year, a longer follow-up would be required for an accurate impact assessment [76]. These results reinforce the idea that adopting a multidisciplinary approach to the care of AKI/AKD survivors may prevent kidney disease progression and its associated outcomes, although longer follow-up studies are required to accurately assess the impact of this approach on clinical outcomes.

The duration of follow-up is associated with long-term patient outcomes, and current evidence supports close monitoring for at least 1 year post-AKI [76–78]. Indeed, follow-up should not be discontinued when GFR returns to normal within 3 months after AKI, as these patients might still be at risk of CKD progression [79].

Current expert consensus recommends that AKD management should follow a comprehensive approach including evaluation of kidney function, review, and adjustment of medication, blood pressure control, and dietary interventions [17, 80]. Additionally, AKI and AKD should be properly listed in patient history.

Kidney function is typically monitored through the assessment of SCr and blood urea nitrogen levels. However, this approach might be misleading in patients with reduced muscle mass, such as critically ill patients, leading to an overestimation of kidney function at the time of discharge and of recovery [81]. Cystatin C may be a better option in these cases. In a retrospective study of 3077 Intensive Care Unit survivors, using cystatin C to calculate estimate GFR at discharge was associated with lower values than using SCr (68 vs 92 ml/min/1.73 m^2^, *P* < .001). Moreover, cystatin C, unlike SCr, was consistently associated with increased 1-year mortality (HR: 1.78, 95% CI 1.46–2.18) [82]. This reinforces the idea that cystatin C may provide a more reliable assessment of kidney function than SCr in AKD patients. Additionally, serum and urinary NGAL could also be useful in evaluating patients at risk for CKD progression [29, 30]. Higher levels of serum and urinary NGAL have been associated with the progression to CKD after AKI in a cohort of 121 critically ill patients (AUC 0.868, sensitivity 77.8%, specificity 91.5%) [83]. Furthermore, reductions of NGAL within 48 hours of admission were also predictive of CKD progression in septic AKI patients. In a single-center study, the reductions of serum and urine NGAL were lower in patients who developed CKD after AKI (*P* < .05) [84].

Quantification of proteinuria is another valuable tool for risk stratification of patients post-AKI. In fact, recent research suggests that proteinuria levels tend to increase following an episode of AKI, potentially reflecting residual renal parenchymal damage and, consequently, an increased risk of subsequent loss of kidney function [85, 86]. According to the ASSESS-AKI prospective cohort study, a higher urine albumin-to-creatinine ratio was associated with an increased risk of kidney disease progression (HR: 1.53 for each doubling; 95% CI 1.45–1.62), and demonstrated strong discriminatory power for its prediction, with a C statistic of 0.82 [87]. These results indicate that routine and widespread quantification of proteinuria after an episode of AKI should be implemented in clinical practice.

Recently, researchers have identified potential new prognostic biomarkers for kidney disease progression after AKI. Following a retrospective study of 1474 hospital survivors from the ASSESS-AKI and ARID studies, plasma levels of soluble tumor necrosis factor receptor 1 (sTNFR1) and sTNFR2 measured 3 months after hospital discharge were independently associated with kidney outcomes, heart failure, and mortality [88]. In line with these findings, another study reported that a multivariable model with sTNFR1, sTNFR2, cystatin C, and estimated GFR, effectively identified patients who declined kidney function in the long term (AUC of 0.79) [89]. Other biomarkers of kidney injury, inflammation, and tubular function, such as KIM-1, chemoattractant protein-1 (MCP-1), uromodulin (UMOD), and epidermal growth factor (EGF), have been the focus of numerous studies as potential novel tools for risk assessment after an episode of AKI. According to a prospective cohort study of 656 hospitalized patients with AKI, increases in urine KIM-1 and MCP-1 from baseline to 12 months were linked to a 2- to 3-fold higher risk of CKD, whereas an increase in UMOD was associated with a 40% reduced risk of CKD [90]. Another study involving 1509 patients from the ASSESS-AKI cohort reported that the combination of urinary EGF/Cr at 3 months with clinical variables demonstrated moderate discrimination for MAKEs (AUC 0.73, 95% CI 0.69–0.77) and strong discrimination for kidney failure at 4 years (AUC 0.96, 95% CI 0.92–1.00) [91]. Taking this into account, these biomarkers might help improve predictive potency for AKI progression. However, the inclusion of these biomarkers into daily clinical practice, remains limited by assay standardization, cost-effectiveness, and lack of widespread validation in real-world settings. Currently, only NGAL has FDA approval and can be used in clinical practice, although it is not widely available. Recently, the FDA has given approval for the confirmatory phase of a panel of eight urinary kidney biomarkers (albumin, total protein, KIM-1, clusterin, cystatin c, NGAL, osteopontin (OPN), and *N*-acetyl-beta-D-glucosaminidase) for AKI detection, which may be of use in monitoring long-term outcomes.

More recently, experts have proposed a multi-biomarker panel, incorporating both serum and urinary biomarkers, to differentiate between the various forms of AKI and AKD, and to predict clinical outcomes [92]. Nevertheless, further studies are required to gain a clearer understanding of which biomarkers should be integrated into clinical practice.

AKD patients should also undergo medication reconciliation and thorough review, as these are essential to enhance recovery and minimize the risk of adverse outcomes [19]. During episodes of acute illness, certain medications, such as non-steroidal anti-inflammatory drugs (NSAIDs), RAASi, diuretics, statins, oral hypoglycemic agents, and sodium-glucose cotransporter 2 inhibitors (SGLT-2i) might need to be withdrawn or adjusted and, after discharge, restarting these medications should be considered as needed [19]. Patient education on withholding nephrotoxic medications is also crucial in this AKD transition phase.

Recent studies have shown promising results regarding the potential benefits of RAASi, mineralocorticoid receptor antagonists (MRAs), as well as SGLT-2i in AKD patients. A recent systematic review reported that a timely initiation of RAASi in AKD patients was associated with lower risk of all-cause mortality (*P* < .01) and adverse kidney events (*P* < .01) [93]. The importance of an early restart of RAASi in selected patients has been emphasized. In a cohort of US veterans with diabetic kidney disease, RAASi reinitiation within 3 months of discharge was associated with reduced mortality and progression to CKD [94]. Moreover, regardless of kidney function recovery, RAASi use after AKI was associated with a significant mortality reduction [94]. Similarly, a recent analysis demonstrated that RAASi resumption within 30 days of hospital discharge after AKI was associated with better renal and cardiovascular outcomes, without increasing the risk of hyperkalemia or rehospitalization for AKI [95]. Thus, nephrologists should consider timely reintroduction of RAASi for patients with proteinuria, heart failure, or diabetic nephropathy.

In a study of 7252 AKD patients with hypertension, MRAs were associated with a lower risk of dialysis in AKD patients (HR: 0.83, 95% CI 0.74–0.93, *P* = .001), although there was an increased risk of hyperkalemia (HR: 1.15, 95% CI 1.04–1.26, *P* = .005) [96]. More recently, an observational study of 230 366 AKD patients with type 2 diabetes reported that medication with SGLT-2i reduced the risk of mortality (HR: 0.69, 95% CI 0.62–0.77), major adverse kidney events (HR: 0.62, 95% CI 0.56–0.69) and major adverse cardiovascular events (HR: 0.75, 95% CI 0.65–0.88) [97]. Taking this into account, clinicians should consider prescribing RAASi and SGLT-2i into the management of AKD patients within 3 months after discharge. It is also important to prescribe appropriate cardiovascular risk prevention medication, namely statins and antiplatelets, according to patients’ medical histories.

In regard to blood pressure control, retrospective data from 43 611 hospitalized patients without a prior history of hypertension suggest that AKI significantly increases the risk of developing elevated blood pressure (OR: 1.22, 95% CI 1.12–1.33), an association that was observed within the first 180 days following hospital discharge and persisted throughout the first 2 years of follow-up [98]. As a result, AKD patients should be closely monitored to ensure optimal blood pressure control, although no guidelines are specified for the blood pressure levels in these patients.

Patients with AKD are at risk of protein-energy malnutrition due to protein catabolism, but also of hyperglycemia, secondary to both peripheral insulin resistance and activation of hepatic gluconeogenesis, and hypertriglyceridemia, caused by an inhibition of lipolysis [99]. Consequently, even though the optimal nutritional therapy for AKD patients remains uncertain, it is imperative to conduct a thorough assessment of their nutritional status and make the appropriate adjustments [17, 80]. Furthermore, given that these patients are more vulnerable to fluid overload, electrolyte abnormalities and acid–base imbalances, it is crucial to carefully monitor their fluid status, salt intake, and electrolytes, with particular attention to foods high in potassium [70]. While evidence supports protein restriction in CKD patients to delay progression and improve metabolic control, its role in AKD is less well defined [100]. Temporary protein restriction may be beneficial by reducing intraglomerular pressure in selected patients, to avoid the risk of malnutrition [99, 101]. Further research is required to clarify the optimal protein targets and its impact on long-term outcomes in AKD patients.

Considering that only a subset of patients will progress to CKD, it is impractical for all patients to be followed up by a nephrologist. A multidisciplinary team including primary care physicians, pharmacists, and dietitians should be involved in post-AKI care.

Moreover, telemedicine may increase the access to nephrology follow-up, especially in rural areas, through virtual consultations and remote monitoring of SCr, GFR, and blood pressure. A pilot study by Silver *et al.* demonstrated the feasibility and acceptability of a nephrology follow-up after AKI via telephone, which had improved follow-up rates, consistent monitoring of kidney function and medication reconciliation, and reduced readmissions when compared to usual care [102].

A risk-stratified follow-up could improve the efficiency of follow-up care by avoiding the need for specialized care for patients with low risk of kidney disease progression, ensuring that all patients are monitored in some capacity following discharge. A six-variable risk prediction tool for advanced CKD after AKI has been developed and externally validated to identify high-risk patients requiring follow-up. This risk score includes clinical and laboratory variables such as age, sex, baseline SCr value, albuminuria, maximum severity of AKI, and SCr value at discharge, and accurately predicted CKD stage 4 within 1 year of discharge (AUC 0.81) [103]. The AFTER AKI trial randomized 155 patients and found that a risk-based intervention increased use of cardioprotective medications among patients at high risk of CKD within 90 days and increased the likelihood of nephrology follow-up among patients with an estimated GFR <30 ml/min per 1.73 m^2^. However, there were no significant differences regarding rates of hospital readmissions, kidney failure, or mortality within 1 year [104].

Finally, to ensure an appropriate follow-up care for these patients, it is essential that they receive a comprehensive and accurate discharge summary, which includes baseline kidney function, peak creatinine and creatinine at discharge, underlying cause of AKI/AKD, patient's hospital course, details regarding intensive care unit admission, need of vasopressors and dialysis requirement, medication recommendations, and a detailed follow-up plan [105].

Cost-effectiveness analyses, although limited, suggest that investment in the follow-up care of these patients may be compensated by reductions in hospital readmissions, dialysis initiation, and long-term morbidity [78, 102, 106, 107]. Addressing these challenges is crucial to improve patient outcomes. Indeed, in a recent analysis of the Tackling AKI study, introducing a treatment approach combining electronic alerts, care bundles and an education program was associated with reduced hospital length of stay and an incremental cost saving of £732 per admission in the UK [106] (Fig. 4). Further cost-effectiveness should be conducted to assess this impact in the long term.

**Figure 3:**
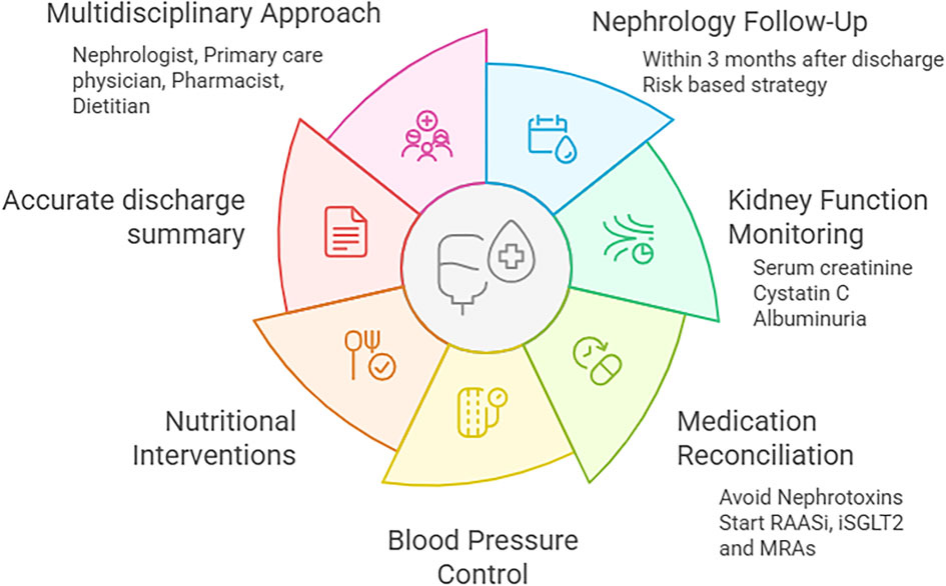
Comprehensive management of AKD patients.

**Figure 4:**
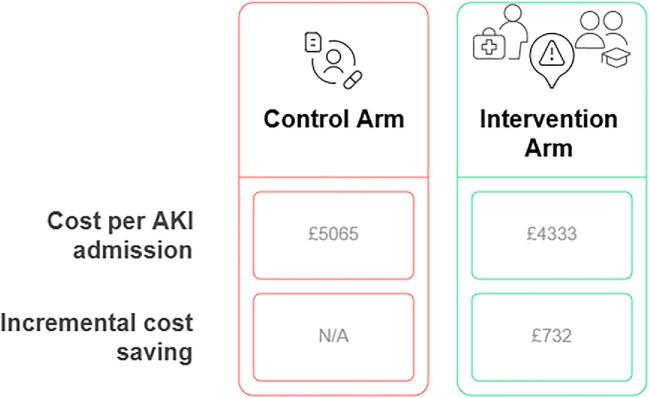
Cost-effectiveness of an AKI intervention strategy. Adapted from Selby *et al.* [106].

## CONCLUSION

Given the increasing prevalence of AKD and its impact on patient prognosis in the long term, it is essential that these patients receive multidisciplinary and effective follow-up care. Considering that current evidence remains scarce to determine which interventions effectively reduce morbidity and mortality in AKD survivors, further randomized controlled clinical trials are of the utmost importance to delineate strategies to halt the AKI-AKD-CKD transition and improve patient outcomes.

## Data Availability

No new data were generated or analyzed in support of this research.
