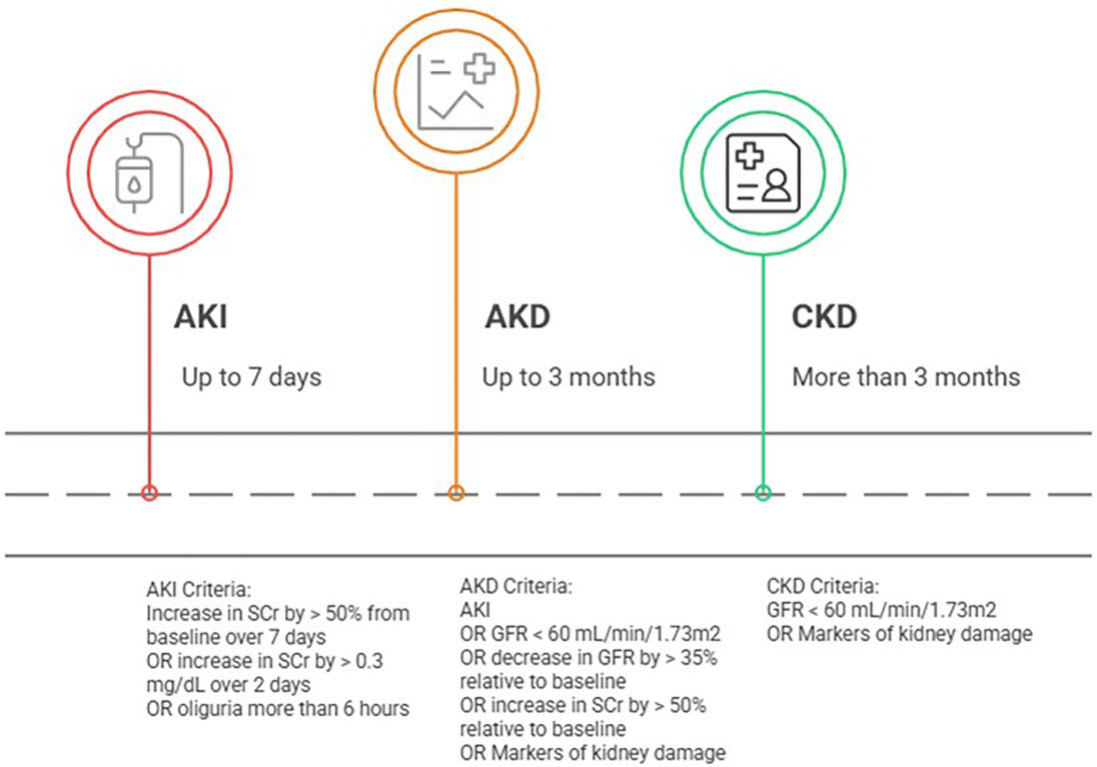# Correction to: The burden of acute kidney disease: an epidemiological review and importance of follow-up care

**DOI:** 10.1093/ckj/sfaf277

**Published:** 2025-09-16

This is a correction to: Joana Gameiro, Beatriz Gouveia, José Agapito Fonseca, José António Lopes, The burden of acute kidney disease: an epidemiological review and importance of follow-up care, *Clinical Kidney Journal*, Volume 18, Issue 6, June 2025, sfaf169, https://doi.org/10.1093/ckj/sfaf169

In figure 1, the text “OR increase in GFR” has been corrected to read “OR decrease in GFR”.

**Figure 1:**